# Industrial Medicine

**Published:** 1920-03-27

**Authors:** 


### Industrial Medicine.

America is paying great attention to the subject of industrial medicine. It is beginning to be felt that this matter concerns the welfare of the nation at large, and that it is not a mere affair between employers and employed.

It is described as "a compromise between the ideals of medicine and the necessities of business," and as a *preventive* measure demands the close attention of the Government.

Another definition is worth quoting: "Industrial medicine is the theory and practice of medicine applied for the purpose of preventing and alleviating the sickness and injury among industrial workers in order that they may enjoy the benefits of continuous productive employment."

Interesting Statistics.

Mr. Selby, the author, in his "Studies of the Medical and Surgical Care of Industrial Workers," also quotes interesting statistics of the number of industrial establishments (155) employing either a whole- or part-time physician. From a study of these we see that in the United States the new movement is under the control of the medical profession, a fact which should stir up the doctors in the United Kingdom to obtain similar facilities for action.

The American movement recognises the need for the physician right the way through industrial life. He is present at the "selection" of employees—the industrial birth—and thus ensures that the right kind of work is found for each person according to his or her physical capacity. He is expected to give his attention to personal ailments, accidents, and tours of the factory, in order to supervise heating, lighting, and ventilation arrangements, and, in short, to give advice in all matters pertaining to the general hygiene of the factory and the health of each worker.

Progress Achieved.

Some progress has been made along these lines in this country, but too much has been left to the goodwill of the more enlightened employers, and until the Government and the medical profession shoulder their responsibilities we cannot hope to advance very far.

What is wanted, says the report, are men "giving their brains and hearts to industry, and striving at all times to better conditions among the working people and to benefit industry itself."

It is interesting to learn that, according to the *Lancet*, a set of forms dealing with industrial health will soon be placed before manufacturers by a Government Department for the United Kingdom.

